# Rotavirus as an Expression Platform of Domains of the SARS-CoV-2 Spike Protein

**DOI:** 10.3390/vaccines9050449

**Published:** 2021-05-03

**Authors:** Asha Ann Philip, John Thomas Patton

**Affiliations:** Department of Biology, Indiana University, Bloomington, IN 47405, USA; aaphilip@iu.edu

**Keywords:** rotavirus, rotavirus vaccine, reverse genetics, *Reoviridae*, expression vector, SARS-CoV-2, COVID-19 vaccine, spike protein

## Abstract

Among vaccines administered to children are those targeting rotavirus, a segmented double-stranded RNA virus that represents a major cause of severe gastroenteritis. To explore the feasibility of establishing a combined rotavirus-SARS-CoV-2 vaccine, we generated recombinant (r)SA11 rotaviruses with modified segment 7 RNAs that contained coding cassettes for NSP3, a translational 2A stop-restart signal, and a FLAG-tagged portion of the SARS-CoV-2 spike (S) protein: S1 fragment, N-terminal domain (NTD), receptor-binding domain (RBD), extended RBD (ExRBD), or S2 core (CR) domain. Generation of rSA11 containing the S1 coding sequence required a sequence insertion of 2.2 kbp, the largest such insertion yet introduced into the rotavirus genome. Immunoblotting showed that rSA11 viruses containing the smaller NTD, RBD, ExRBD, and CR coding sequences expressed S-protein products of expected size, with ExRBD expressed at highest levels. These rSA11 viruses were genetically stable during serial passage. In contrast, the rSA11 virus containing the full-length S coding sequence (rSA11/NSP3-fS1) failed to express its expected 80 kDa fS1 product, for unexplained reasons. Moreover, rSA11/NSP3-fS1 was genetically unstable, with variants lacking the S1 insertion appearing during serial passage. Nonetheless, these results emphasize the potential usefulness of rotavirus vaccines as expression vectors of immunogenic portions of the SARS-CoV-2 S protein, including NTD, RBD, ExRBD, and CR, that have sizes smaller than the S1 fragment.

## 1. Introduction

The impact of severe acute respiratory syndrome coronavirus 2 (SARS-CoV-2) on human mortality and morbidity has stimulated-broad ranging efforts to develop vaccines preventing coronavirus disease 19 (COVID-19) [1,2]. Given that the virus can cause asymptomatic and symptomatic infections in individuals of all ages, comprehensive strategies to control the SARS-CoV-2 pandemic may require modification of childhood immunization programs to include COVID-19 vaccines [3,4]. Among the vaccines routinely administered to infants in the US and many other countries are those targeting rotavirus, a segmented double-stranded (ds)RNA virus that is a primary cause of severe acute gastroenteritis (AGE) in children during the first 5 years of life [5,6,7]. The most widely used rotavirus vaccines are given orally and formulated from live-attenuated virus strains [8]. These vaccines induce the production of neutralizing IgG and IgA antibodies [9,10,11] and have been highly effective in reducing the incidence of rotavirus hospitalizations and mortality [12,13].

Advances in rotavirus reverse genetics technologies have allowed the generation of recombinant rotaviruses that serve as expression platforms of heterologous proteins [14,15,16,17,18,19,20,21,22]. The rotavirus genome consists of 11 segments of dsRNA, with a total size of ~18.6 kbp for group A strains (rotavirus species A) typically associated with pediatric AGE [23]. Except for segment 11, all the rotavirus genome segments contain a single open-reading frame (ORF); these encode the six structural (VP1–VP4, VP6–VP7) or six non-structural (NSP) viral proteins [24]. The recently-developed rotavirus reverse genetics systems consist of eleven T7 transcription (pT7) vectors, each directing synthesis of a unique viral single-stranded (+)RNA when transfected into baby-hamster kidney cells producing T7 RNA polymerase (BHK-T7 cells). In some cases, support plasmids expressing capping enzymes [African swine fever virus NP868R [25] or vaccinia virus D1L/D12R [15]] or fusion proteins [avian reovirus p10FAST [15]] are co-transfected with the pT7 vectors to enhance recovery of recombinant viruses. Rotavirus reverse genetics systems have been used to mutate several of the viral genome segments and to generate virus strains that express reporter proteins [17,19,26,27,28,29,30].

Genome segment 7 of group A rotaviruses encodes NSP3 (36 kDa), an RNA-binding protein that acts a translation enhancer of viral (+)RNAs and is expressed at moderate levels in infected cells [31,32]. In a previous study, we showed that the single NSP3 ORF could be re-engineered by reverse genetics to express two separate proteins through placement of a porcine teschovirus 2A translational stop-restart element at the end of the NSP3 ORF, followed by the coding sequence for a heterologous protein [19]. Through this approach, well-growing genetically stable recombinant rotaviruses have been generated that express NSP3 and one or more fluorescent proteins (FPs) [e.g., mRuby (red), UnaG (green), and TagBFP (blue)] from segment 7, an advance allowing study of rotavirus biology by live cell imaging [19,20]. The NSP3 product of these recombinant viruses is functional, capable of dimerization and inducing the nuclear accumulation of the cellular poly(A)-binding protein [19,21]. Thus, recombinant rotaviruses that express foreign proteins via addition of a 2A element and coding sequence into segment 7 downstream of the NSP3 ORF retain the full complement of functional viral ORFs.

**Figure 1:**
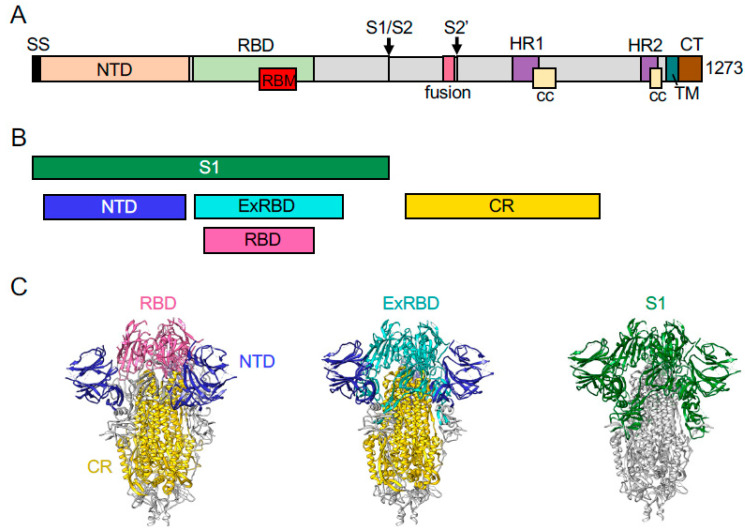
Domains of the SARS-CoV-2 S protein expressed by rSA11. (**A**) S protein trimers are cleaved at the S1/S2 junction by furin proconvertase and at the S2′ site by the TMPRSS2 serine protease. The S1 fragment contains a signal sequence (SS), N-terminal domain (NTD), receptor-binding domain (RBD), receptor-binding motif (RBM), coiled-coil (CC), and two heptad repeats (HR1, HR2). The S2 fragment contains a trimeric core region, transmembrane anchor (TM), and fusion domain. (**B**) Portions of the S protein expressed by recombinant rotaviruses are indicated. (**C**) Ribbon representations of the closed conformation of the trimeric S protein (PDB 6VXX) showing locations of the RBD (magenta), extended RBD (ExRBD, cyan), NTD (blue), core (CR, gold) domains and the S1 cleavage product (green).

As a step towards developing a combined rotavirus-SARS-CoV-2 vaccine, we explored the possibility of generating recombinant rotaviruses that express regions of the SARS-CoV-2 spike (S) protein through re-engineering of the NSP3 ORF in segment 7. Trimers of the S protein form crown-like projections that emanate from the lipid envelop surrounding the SARS-CoV-2 virion [33,34]. Cleavage of the trimeric spikes by furin-like proteases generates S1 and S2 fragments, each of which possesses activities essential for virus entry (Figure 1). The S1 fragment includes an N-terminal domain (NTD) and a receptor-binding domain (RBD), the latter mediating virus interaction with the cell surface receptor angiotensin-converting enzyme 2 (ACE2) [35,36]. The S2 fragment is responsible for S-protein trimerization and contains fusion domains that are essential for virus entry. SARS-CoV-2-specific antibodies with neutralizing activity have been mapped to various regions of the S protein, including the NTD, RBD, and fusion domains [37,38,39,40,41,42,43,44]. We determined that by inserting S coding sequences into rotavirus genome segment 7 downstream of the NSP3 ORF and a 2A element, well-growing genetically stable recombinant rotaviruses can be made that express domains of the S1 and S2 fragments. These findings raise the possibility of constructing rotavirus vaccine strains that are not only capable of inducing immunological protective responses against rotavirus, but also COVID-19.

## 2. Materials and Methods

### 2.1. Cell Culture

Embryonic monkey kidney cells (MA104) were grown in medium 199 (M199) containing 5% fetal bovine serum (FBS) and 1% penicillin-streptomycin. Baby hamster kidney cells expressing T7 RNA polymerase (BHK-T7) were provided by Dr. Ulla Buchholz, Laboratory of Infectious Diseases, NIAID, NIH, and were propagated in Glasgow minimum essential media (GMEM) containing 5% heat-inactivated fetal bovine serum (FBS), 10% tryptone-peptide broth, 1% penicillin-streptomycin, 2% non-essential amino acids, and 1% glutamine [45]. BHK-T7 cells were grown in medium supplemented with 2% Geneticin (Invitrogen, Thermo Fisher Scientific, Waltham, MA, USA) with every other passage. 

### 2.2. Plasmid Construction

Recombinant SA11 rotaviruses were prepared using the plasmids pT7/VP1SA11, pT7/VP2SA11, pT7/VP3SA11, pT7/VP4SA11, pT7/VP6SA11, pT7/VP7SA11, pT7/NSP1SA11, pT7/NSP2SA11, pT7/NSP3SA11, pT7/NSP4SA11, and pT7/NSP5SA11 [https://www.addgene.org/Takeshi_Kobayashi/, accessed on: 28 April 2021] and pCMV-NP868R [15]. The plasmid pT7/NSP3-P2A-fUnaG was produced, as described elsewhere, by fusing a DNA fragment containing the ORF for P2A-3xFL-UnaG to the 3′-end of the NSP3 ORF in pT7/NSP3SA11 [46]. A plasmid (pTWIST/COVID19spike) containing a full-length cDNA of the SARS-CoV-2 S gene (GenBank MN908947.3) was purchased from Twist Bioscience (South San Francisco, CA, USA). The plasmids pT7/NSP3-2A-fNTD, pT7/NSP3-2A-fExRBD, pT7/NSP3-2A-fRBD, pT7/NSP3-2A-fCR, and pT7/NSP3-2A-S1 were made by replacing the UnaG ORF in pT7/NSP3-2A-fUnaG with ORFs for the NTD, ExRBD, RBD, CR, and S1 regions, respectively, of the SARS-CoV-2 S protein, using an In-Fusion cloning kit (Takara Bio USA, Mountain View, CA, USA). DNA fragments containing NTD, ExRBD, RBD, CR, and S1 coding sequences were amplified from pTWIST/COVID19spike using the primer pairs NTD_For and NTD_Rev, ExRBD_For and ExRBD_Rev, RBD_For and RBD_Rev, CR_For and CR_Rev, and S1_For and S1_Rev, respectively (Table 1). Transfection quality plasmids were prepared commercially (www.plasmid.com, accessed on: 28 April 2021) or using Qiagen plasmid purification kits (Qiagen, Germantown, MD, USA). Primers were provided by and sequences determined by Eurofins Genomics (Louisville, KY, USA).

### 2.3. Recombinant Viruses

The reverse genetics protocol used to generate recombinant rotaviruses was described in detail previously [21,46]. To summarize, BHK-T7 cells were transfected with SA11 pT7 plasmids and pCMV-NP868R using Mirus TransIT-LT1 transfection reagent (Mirus Bio, Madison, WI, USA). Two days later, the transfected cells were overseeded with MA104 cells and the growth medium (serum-free) adjusted to a final concentration of 0.5 µg/mL porcine Type IX pancreatic trypsin (Sigma Aldrich, St. Louis, MO, USA) [45]. Three days later, the BHK-T7/MA104 cell mixture was freeze–thawed 3-times and the lysates clarified by low-speed centrifugation. Recombinant virus in clarified lysates were amplified by one or two rounds of passage in MA104 cells maintained in serum-free medium containing 0.5 µg/mL trypsin. Individual virus isolates were obtained by plaque purification and typically amplified 1 or 2 rounds in MA104 cells prior to analysis. Viral dsRNAs were recovered from infected-cell lysates by Trizol extraction (Thermo Fisher Scientific), resolved by electrophoresis on Novex 8% polyacrylamide gels (Thermo Fisher Scientific) in Tris-glycine buffer, and detected by staining with ethidium bromide. Viral dsRNAs in gels were visualized using a Bio-Rad ChemiDoc MP Imaging System (Bio-Rad Laboratories, Hercules, CA, USA). The genetic stability of plaque isolated rSA11s was assessed by serial passage as described previously [19].

### 2.4. Immunoblot Analysis

MA104 cells were mock infected or infected with 5 PFU of recombinant virus per cell and harvested at 8 h p.i. Cells were washed with cold phosphate-buffered saline (PBS), pelleted by low-speed centrifugation, and lysed by resuspending in lysis buffer [300 mM NaCl, 100 mM Tris-HCl, pH 7.4, 2% Triton X-100, and 1x ethylenediaminetetraacetic acid (EDTA)-free protease inhibitor cocktail (Roche cOmplete, Sigma Aldrich)]. For immunoblot assays, lysates were resolved by electrophoresis on Novex linear 8–16% polyacrylamide gels and transferred to nitrocellulose membranes. After blocking with phosphate-buffered saline containing 5% non-fat dry milk, blots were probed with guinea pig polyclonal NSP3 (Lot 55068, 1:2000 dilution) or VP6 (Lot 53963, 1:2000) antisera [47], mouse monoclonal FLAG M2 (F1804, Sigma-Aldrich, 1:2000), rabbit monoclonal PCNA [13110S, Cell Signaling Technology (CST), Danvers, Massachusetts, USA), 1:1000] antibody or rabbit anti-RBD (ProSci 9087, Poway, California, USA, 1:200) antibody. Primary antibodies were detected using 1:10,000 dilutions of horseradish peroxidase (HRP)-conjugated secondary antibodies: horse anti-mouse IgG (CST), anti-guinea pig IgG [Kirkegaard & Perry Laboratories (KPL), SeraCore Life Sciences, Gaithersburg, Maryland, USA)], or goat anti-rabbit IgG (CST). Signals were developed using Clarity Western ECL Substrate (Bio-Rad) and detected using a Bio-Rad ChemiDoc imaging system.

### 2.5. Immunoprecipitation Assay

Mock-infected and infected cell lysates were prepared as above. Lysates were mixed with a SARS-CoV-2 S1 specific monoclonal antibody (CR3022, GeneTex, Irvine, California, USA, 1:150 dilution) or an NSP2 monoclonal antibody (#171, 1:200). After incubation at 4 °C with gentle rocking for 18 h, antigen-antibody complexes were recovered using Pierce magnetic IgA/IgG beads (Thermo Fisher Scientific), resolved by gel electrophoresis, and blotted onto nitrocellulose membranes. Blots were probed with FLAG antibody (1:2000) to detect fRBD and fExRBD and NSP2 antibody (1:2000).

### 2.6. CsCl Gradient Centrifugation

MA104-cell monolayers in 10 cm cell culture plates were infected with rSA11 viruses at an MOI of 5 and harvested at 12 h p.i. Cells were lysed by adjusting media to 0.5% Triton X100 (Sigma) and incubation on ice for 5 min. Lysates were then clarified by centrifugation at 500× *g* at 4 °C for 6 min. The clarified lysates were adjusted to 10 mM EDTA and incubated for 1 h at 37 °C to cause the conversion of rotavirus TLPs to DLPs [45]. CsCl was added to samples to a density of 1.367 g/cm^3^ and samples were centrifuged at 110,000× *g* with a Beckman SW55Ti rotor (Beckman Coulter Life Sciences, Indianapolis, IN, USA) at 8 °C for 22 h. Fractions containing viral bands were recovered using a micropipettor and fraction densities were determined using a refractometer.

### 2.7. Genetic Stability of rSA11 Viruses

Viruses were serially passaged on MA104-cell monolayers using 1:1000 dilutions of infected cell lysates prepared in serum-free M199 medium and 0.5 µg/mL trypsin. When cytopathic effects reached completion (4–5 days), cells were freeze–thawed twice in their medium, and lysates were clarified by low-speed centrifugation. To recover dsRNA, clarified lysates (600 µL) were extracted with Trizol (Thermo Fisher Scientific). The RNA samples were resolved by electrophoresis on 8% polyacrylamide gels and the bands of dsRNA detected by ethidium-bromide staining.

### 2.8. GenBank Accession Numbers

Segment 7 sequences in rSA11 viruses have been deposited in Genbank: wt (LC178572), NSP3-P2A-fNTD (MW059024), NSP3-P2A-fRBD (MT655947), NSP3-P2A-ExRBD (MT655946), NSP3-P2A-fCR (MW059025), NSP3-P2A-S1 (MW059026), NSP3-P2A-S1/R1 (MW353715), NSP3-P2A-S1/R2 (MW353716), and NSP3-P2A-S1/R3 (MW353717). See also Table 2.

## 3. Results and Discussion

### 3.1. Modified Segment 7 (NSP3) Expression Vectors Containing SARS-CoV-2 S Sequences

**Figure 2:**
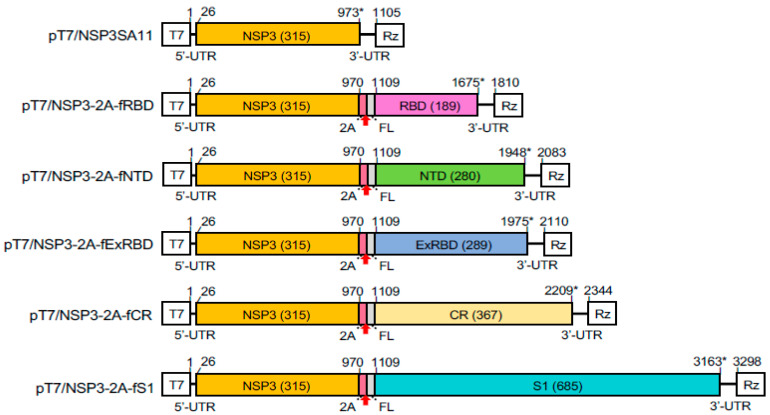
Plasmids with modified segment 7 (NSP3) cDNAs used to generate rSA11 viruses expressing regions of the SARS-CoV-2 S protein. Illustration indicates nucleotide positions of the coding sequences for NSP3, porcine teschovirus 2A element, 3xFLAG (FL), and the complete S1 or portions of the S1 (NTD, ExRBD, and RBD) and S2 (CR) proteins. The red arrow notes the position of the 2A translational stop-restart site, and the asterisk (*) notes the end of the ORF. Sizes (aa) of encoded NSP3 and S products are in parenthesis. T7 (T7 RNA polymerase promoter sequence), Rz (Hepatitis D virus ribozyme), and UTR (untranslated region).

To examine the possibility of using rotavirus as an expression platform for regions of the SARS-CoV-2 S protein, we replaced the NSP3 ORF in the pT7/NSP3SA11 transcription vector with a cassette comprised of the NSP3 ORF, a porcine teschovirus 2A element, and a coding sequence of the S protein (Figure 2). The cassette included a flexible GAG hinge between the coding sequence for NSP3 and the 2A element and a 3x FLAG (f) tag between the coding sequences for the 2A element and the S region. This approach was used to generate a set of vectors (collectively referred to as pT7/NSP3-CoV2/S vectors) that contained coding sequences for SARS-CoV-2 S1 (pT7/NSP3-2A-fS1), NTD (pT7/NSP3-2A-fNTD), RBD (pT7/NSP3-2A-fRBD), an extended form of the RBD (ExRBD) (pT7/NSP3-2A-fExRBD), and the S2 core region (CR) including its fusion domains (pT7/NSP3-2A-fCR) (Figure 1). The S sequences were inserted into the pT7/NSP3SA11 vector at the same site as used before in the production of recombinant SA11 (rSA11) rotaviruses expressing FPs [19,21].

### 3.2. Recovery of rSA11 Rotaviruses with Segment 7 dsRNA Containing S Sequences

To generate rSA11 viruses, BHK-T7 monolayers were transfected with a complete set of pT7/SA11 expression vectors, except pT7/NSP3SA11 was replaced with a pT7/NSP3-CoV2/S vector, and a CMV expression plasmid (pCMV-NP868R) encoding the capping enzyme of African swine fever virus. In transfection mixtures, plasmids encoding rotavirus NSP2 (pT7/NSP2SA11) and NSP5 (pT7/NSP5SA11) were included at levels three-fold greater than the other pT7/SA11 vectors. BHK-T7 cells were overseeded with MA104 cells two days following transfection. The BHK-T7/MA104 cell mixture was freeze–thawed three days later, and the rSA11 viruses were recovered by plaque isolation and amplified by 1 or 2 cycles of growth in MA104 cells prior to characterization [45]. Properties of the rSA11 viruses are summarized in Table 2.

**Figure 3:**
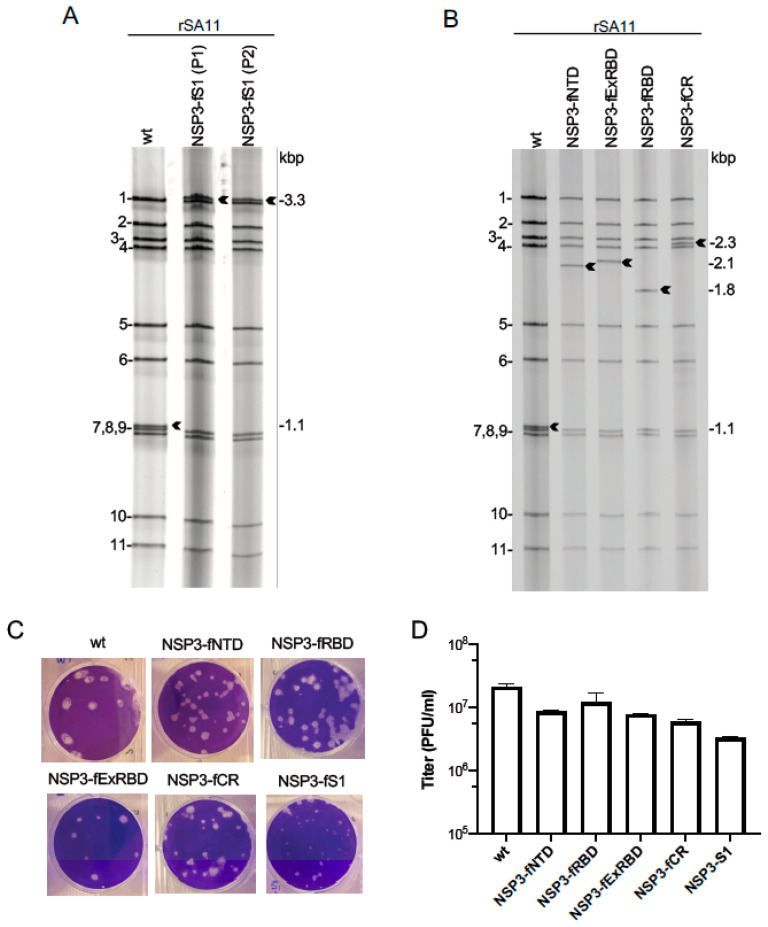
Properties of rSA11/NSP3-CoV2/S viruses expressing regions of the SARS-CoV-2 S protein. (**A**,**B**) dsRNA was recovered from MA104 cells infected with plaque-purified rSA11 isolates, resolved by gel electrophoresis, and detected by ethidium-bromide staining. RNA segments of rSA11/wt are labeled 1 to 11. Sizes (kbp) of segment 7 RNAs (black arrows) of rSA11 isolates are indicated. Double-stranded RNA of rSA11/NSP3-fS1 serially passaged twice (P1 and P2) in MA104 cells is shown in (**A**). (**C**) Plaque assays were performed using MA104 cells and detected by crystal-violet staining. (**D**) Titers reached by rSA11 isolates were determined by plaque assay. Bars indicate standard deviations calculated from three separate determinations.

Based on gel electrophoresis, rSA11 viruses generated with pT7/NSP3-S vectors (collectively referred to as rSA11/NSP3-CoV2/S viruses) contained segment 7 dsRNAs that were much larger than that of wild-type rSA11 (rSA11/wt) virus (Figure 3). Sequence analysis confirmed that the segment 7 dsRNAs of the rSA11/NSP3-CoV2/S viruses matched the segment 7 sequences present in the pT7/NSP3-CoV2/S vectors (data not shown). The re-engineered segment 7 dsRNA of virus isolate rSA11/NSP3-fS1 had a length of 3.3 kbp, accounting for its electrophoretic migration near the largest rotavirus genome segment (segment 1), which is likewise 3.3 kbp in length (Table 2, Figure 3A). The segment 7 dsRNA of rSA11/NSP3-fS1 contains a 2.2 kbp foreign sequence insertion, the longest foreign sequence that has been introduced into the segment 7 dsRNA, or for that matter, any rotavirus genome segment. The previously longest 7 dsRNA engineered into rSA11 was the 2.4 kbp segment 7 dsRNA of rSA11/NSP3-fmRuby-P2A-fUnaG, which contained a cassette that encoded three proteins (NSP3, UnaG, mRuby) [19]. The total genome size of rSA11/NSP3-fS1 is 20.8 kbp, 12% greater than that of rSA11/wt [49]. This is the largest genome known to exist within a rotavirus isolate and demonstrates the capacity of rotavirus to replicate and package large amounts of foreign sequence.

The segment 7 dsRNAs of virus isolates, rSA11/NSP3-fNTD, -fRBD, -fExRBD, and -fCR, were determined to have lengths of 2.1, 1.8, 2.1, and 2.3 kbp, respectively (Table 2), and as expected from their sizes, migrated on RNA gels between rotavirus genome segments 3 (2.6 kbp) and 5 (1.6 kbp) (Figure 3B). The segment 7 dsRNAs of the rSA11/NSP3-fNTD, -fRBD, -fExRBD, and -fCR isolates contained foreign sequence insertions of 1.0, 0.7, 1.0, and 1.2 kbp, respectively, significantly smaller that the 2.1 kbp foreign sequence insertion of rSA11/NSP3-fS1. The smaller sizes of the foreign-sequence inserts contained in the segment 7 RNAs of rSA11/NSP3-fNTD, -fRBD, -fExRBD, and -fCR may provide the additional genetic space necessary for re-engineering the S-protein products of these viruses to include routing and localization tags capable of enhancing antigen recognition and processing by immune cells. Particularly valuable may be the inclusion of tags that promote interaction of the S-protein products with antibody heavy-chain (Fc) receptors (e.g., FcRn) [50], enable aggregation or multivalent presentation of the products [51], or increase the efficiency of synthesis or secretion of the products [52,53,54].

Consistent with previous studies examining the phenotypes of rSA11 isolates expressing FPs [19,21], the sizes of plaques formed by rSA11/NSP3-CoV2/S viruses were smaller than plaques formed by rSA11/wt (Figure 3C). Similarly, rSA11 viruses containing S-protein coding sequences grew to maximum titers that were up to 0.5–1 log lower than rSA11/wt (Figure 3D). The reason for the smaller plaques and lower titers of the rSA11/NSP3-CoV2/S viruses is unknown, but may reflect the longer elongation time likely required for the viral RNA polymerase to transcribe their segment 7 dsRNAs during viral replication. Alternatively, it may reflect the longer time required to translate segment 7 (+)RNAs that contain S-protein coding sequences.

### 3.3. Expression of S Coding Sequences by rSA11 Rotaviruses

**Figure 4:**
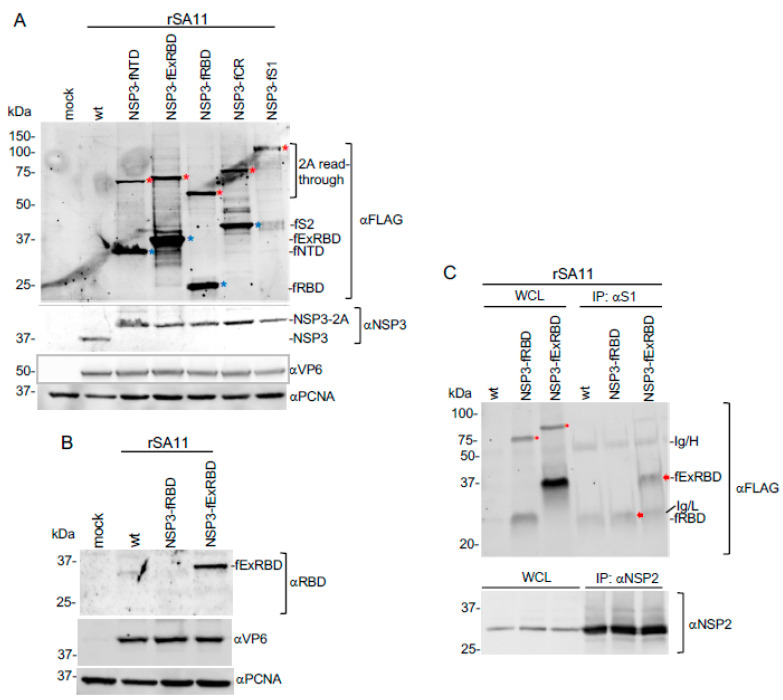
Expression of SARS-CoV-2 S products by rSA11 viruses. (**A**,**B**) Whole cell lysates (WCL) were prepared from cells infected with rSA11 viruses and examined by immunoblot assay using (**A**) FLAG antibody to detect S products (NTD, ExRBD, RBD, CR, S1, and 2A read-through products) and antibodies specific for rotavirus NSP3 and VP6 and proliferating cell nuclear antigen (PCNA). Red asterisks (*) identify 2A read-through products and blue asterisks (*) identify 2A cleavage products. Cleaved fS1 product failed to be detected in five separate immunoblot assays analyzing two independently generated samples of MA104 cells infected with rSA11/NSP3-fS1 virus. (**B**) Lysates prepared from MA104 cells infected with rSA11wt, rSA11/NSP3-fRBD and rSA11/NSP3-fExRBD were examined by immunoblot assay using antibodies specific for RBD (ProSci 9087), rotavirus VP6, and PCNA. (**C**) Lysates prepared from MA104 cells infected with rSA11/wt, rSA11/NSP3-fRBD and rSA11/NSP3-fExRBD viruses were examined by immunoprecipitation assay using a SARS-CoV-2 S1 specific monoclonal antibody (GeneTex CR3022). Lysates were also analyzed with a NSP2-specific polyclonal antibody. Antigen-antibody complexes were recovered using IgA/G beads, resolved by gel electrophoresis, blotted onto nitrocellulose membranes, and probed with FLAG (fRBD and fExRBD) and NSP2 antibody. Molecular weight markers are indicated (kDa). Red arrows indicate fRBD and fExRBD. fRBD comigrates near the Ig light chain (Ig/L). Ig heavy chain, Ig/H).

To determine whether the rSA11/NSP3-CoV2/S viruses expressed products from their S sequences, lysates prepared from MA104 cells infected with these viruses were examined by immunoblot assay using FLAG- and RBD-specific antibodies (Figure 4A,B). Immunoblots probed with FLAG antibody showed that rSA11/NSP3-fNTD, -fExRBD, -fRBD, and -fCR viruses generated S products and that their sizes were as predicted for an active 2A element in the segment 7 ORF: fNTD (34.8 kDa), fExRBD (35.2 kDa), fRBD (24.3 kDa), and fCR (42.9 kDa) (Table 2). Immunoblot assays indicated that the rSA11/NSP3-fExRBD yielded higher levels of S product than any of the other rSA11/NSP3-CoV2/S viruses. The basis for the higher levels of the fExRBD product is unclear, but does not correlate with increased levels of expression of other viral products, such as NSP3 and VP6. Nonetheless, the high levels of ExRBD expression by rSA11/NSP3-fExRBD suggests that such viruses may be best suited in pursing the development of combined rotavirus/COVID vaccines.

FLAG antibody did not detect the expected 79.6 kDa fS1 product in cells infected with rSA11/NSP3-fS1 (Figure 4A). The explanation for this is unknown, may relate to modifications made to the S protein during its synthesis. Notably, the S1 coding sequence in the segment 7 ORF includes an N-terminal signal sequence which, in SARS-CoV-2 infected cells, is cleaved from the S1 protein during synthesis on the endoplasmic reticulum (ER) [33,55]. Cleavage of the signal sequence may have removed the upstream 3x FLAG tag from a S1 product, preventing its detection by the FLAG antibody. Alternatively, because the membrane anchor domain is located at the C-terminus of the SARS-CoV-2 S protein, it is possible that the S1 product was secreted from rSA11/NSP3-fS1-infected cells and thus lost [56]. It is also possible that glycosylation and/or degradation of the 79.6 kDa-S1 product by ER-associated proteases may have prevented the protein’s detection. Finally, because rotavirus usurps and possibly remodels the ER in support of glycoprotein (NSP4 and VP7) synthesis and virus morphogenesis may perturb ER interaction with the S signal sequence in such a way to prevent S1 synthesis [24].

Interestingly, all the rSA11/NSP3-CoV2/S viruses, including rSA11/NSP3-fS1, generated 2A read-through products that were detectable using FLAG antibody (Figure 4A). Thus, the 2A stop-start element in the rSA11/NSP3-2A-CoV2/S viruses was not fully active, which is consistent with previous reports analyzing the functionality of 2A elements within cells [57,58,59]. However, with the exception of the rSA11/NSP3-fS1, all the viruses generated more 2A-cleaved S product than read-through product. Mutation of residues in and around the 2A element, including the inclusion of flexible linker sequences, may decrease the relative frequency of read through [60,61].

Lysates from MA104 cells infected with rSA11/wt, rSA11/NSP3-fRBD, and rSA11/NSP3-fExRBD were also probed with a RBD-specific polyclonal antibody prepared against a peptide mapping to the C-terminal end of the RBD domain (ProSci 9087). The RBD antibody recognized the fExRBD product of the rSA11/NSP3-fExRBD virus, but not the fRBD product of rSA11/NSP3-fRBD (Figure 4B), presumably because the latter product lacked the peptide sequence used in generating the ProSci RBD antibody. To gain insight into whether the fRBD and fExRBD products folded into native structures mimicking those present in the SARS-CoV-2 S protein, lysates prepared from MA104 cells infected with rSA11/NSP3-fRBD and rSA11/NSP3-fExRBD were probed by pulldown assay using an anti-RBD conformation-dependent neutralizing monoclonal antibody (GeneTex CR3022) [62]. As shown in Figure 4C, the CR3022 immunoprecipitate included fExRBD, indicating that this product included a neutralizing epitope found in authentic SARS-CoV-2 S protein. Thus, at least some of the RBD product of rSA11/NSP3-fExRBD has likely folded in a conformation capable of inducing a protective antibody response. Unlike the successful pulldown of ExRBD with CR3022 antibody, it was not clear if the antibody likewise immunoprecipitated the fRBD product of rSA11/NSP3-fRBD. This uncertainty stems from the light chain of the CR3022 antibody obscuring the electrophoretically closely-migrating fRBD product in immunoblot assays (Figure 4C).

### 3.4. Expression of the ExRBD and RBD Products by rSA11s during Rotavirus Infection

**Figure 5:**
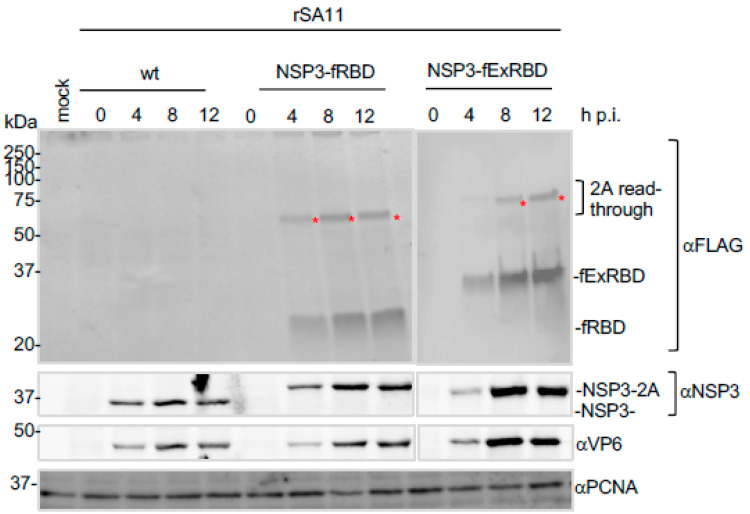
Production of RBD and ExRBD by rSA11 viruses during infection. MA104 cells were mock infected or infected with rSA11/wt, rSA11/NSP3-fRBD, or rSA11/NSP3-fExRBD (MOI of 5). Lysates were prepared from the cells at 0, 4, 8, or 12 h p.i. and analyzed by immunoblot assay using antibodies specific for FLAG, NSP3, VP6, and PCNA. Red asterisks (*) identify 2A read-through products. Positions of molecular weight markers are indicated (kDa).

To gain insight into fExRBD and fRBD expression during virus replication, MA104 cells were infected with rSA11/wt, rSA11/NSP3-fExRBD or rSA11/NSP3-fRBD and then harvested at intervals between 0 and 12 h p.i. Analysis of the infected cell lysates by immunoblot assay showed that fExRBD and fRBD were readily detectable by 4 h p.i., paralleling the expression of rotavirus proteins NSP3 and VP6 (Figure 5). Increased levels of fExRBD and fRBD were present at 8 and 12 h p.i., without obvious accumulation of FLAG-tagged products of smaller sizes. Thus, the fExRBD and fRBD products appear to be relatively stable.

### 3.5. Density of rSA11 Virus Particles Containing S Sequences

**Figure 6:**
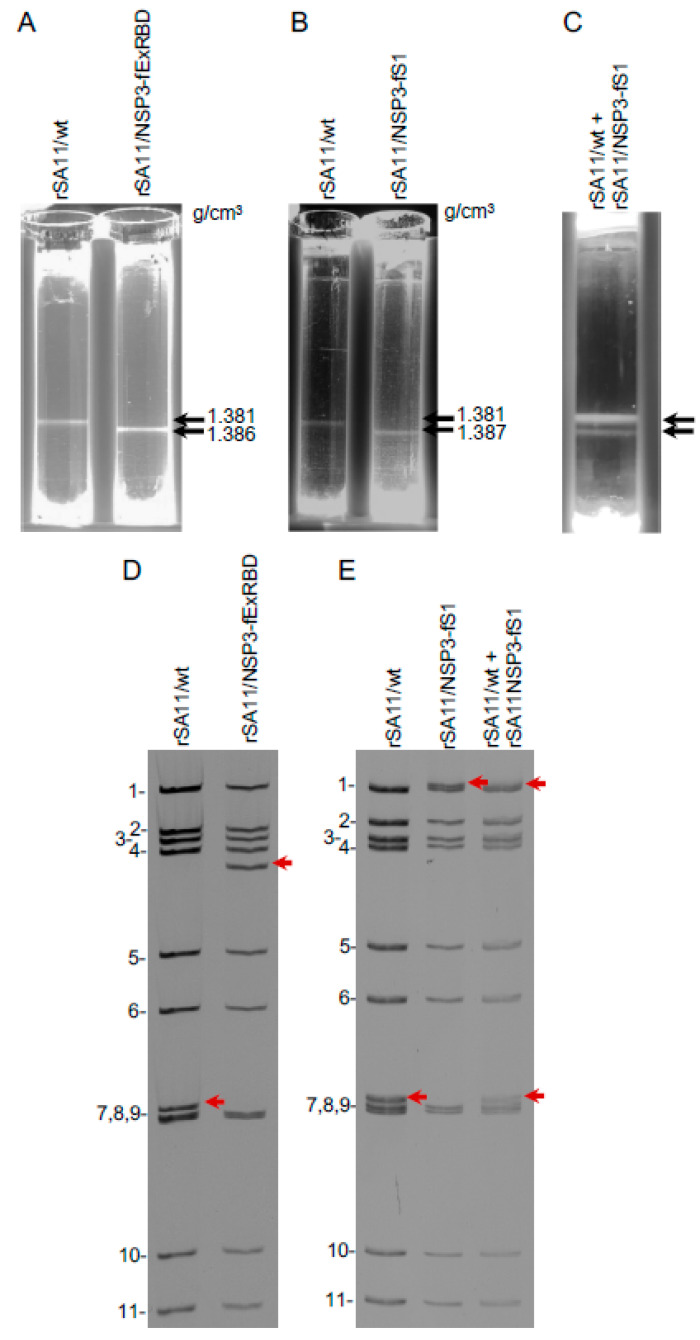
Impact of genome size on rotavirus particle density. MA104 cells were infected with rSA11/wt, rSA11/NSP3-fExRBD, or rSA11/NSP3-fS1 viruses at an MOI of 5. At 12 h p.i., the cells were recovered, lysed by treatment with non-ionic detergent, and treated with EDTA to convert rotavirus virions into DLPs. (**A**,**B**) DLPs were banded by centrifugation in CsCl gradients and densities (g/cm^3^) were determined using a refractometer. (**C**) Lysates from rSA11/wt and rSA11/NSP3-fS1 infected cells were combined and their DLP components banded by centrifugation in a CsCl gradient. (**D**,**E**) Electrophoretic profile of the dsRNA genomes of DLPs recovered from CsCl gradients. Panel D RNAs derive from DLPs in panel A and panel E RNAs derive from DLPs in panel B and C. RNA segments of rSA11/wt are labeled 1 to 11. Positions of segment 7 RNAs are indicated with red arrows.

The introduction of S sequences into the rSA11/NSP3-CoV2/S viruses increased the size of their viral genomes by 1.0 to 2.5 kbp beyond that of SA11/wt. Assuming the rSA11/NSP3-CoV2/S viruses are packaged efficiently and contain a complete constellation of 11 genome segments, the increased content of dsRNA within the core of rSA11/NSP3-CoV2/S particles should cause their densities to be greater than that of SA11/wt particles. To explore this possibility, rSA11/wt (18.6 kbp genome), rSA11/NSP3-fExRBD (19.5 kbp) and rSA11/NSP3-fS1 (20.8 kbp) were amplified in MA104 cells. The infected-cell lysates were then treated with EDTA to convert rotavirus virions (triple-layered particles) into double-layered particles (DLPs). The particles were centrifuged to equilibrium on CsCl gradients and the density of the DLP bands determined by refractometry (Figure 6). The analysis indicated that the density of rSA11/NSP3-fExRBD DLPs (1.386 g/cm^3^) was greater than SA11/wt DLPs (1.381 g/cm^3^) (panel A) and similarly, the density of rSA11/NSP3-fS1 DLPs (1.387 g/cm^3^) was greater that SA11/wt DLPs (1.38 g/cm^3^) (panel B). Analysis of the banded DLPs by gel electrophoresis confirmed that they contained the expected constellation of eleven genome segments. To confirm that the density of rSA11/NSP3-fS1 DLPs was different to rSA11/wt DLPs, infected-cell lysates containing each of these viruses were pooled, treated with EDTA, and the viral DLPs in the combined sample banded by centrifugation on a CsCl gradient (Figure 6E). Analysis of the gradient revealed the presence of two bands of particles, indicating that rSA11/NSP3-fSA11-fS1 and rSA11/wt DLPs were of different densities. Gel electrophoresis of the combined DLP bands showed, as expected, that both rSA11/NSP3-fSA11-fS1 and rSA11/wt were present. Taken together, these results demonstrate that rSA11/NSP3-CoV-2/S virions contain complete genome constellations despite the fact that their genome sizes are significantly greater than that of wild-type SA11 virus. Indeed, the 20.8 kbp rSA11/NSP3-fS1 genome is 12% greater in size than the 18.6 kbp rSA11/wt genome (Table 2). Thus, the rotavirus core has space to accommodate large amounts of additional foreign sequence. How the dsRNA within the core is re-distributed to accommodate large amounts of additional sequence is not known, but clearly the core remains a transcriptionally-active nanomachine despite the additional sequence. Whether other genome segments can be engineered similarly to segment 7 of rSA11/NSP3-fS1 to include 2 kb of additional sequence remains to be determined. The maximum packaging capacity of the core also remains to be determined. Our findings are consistent with earlier studies showing that the density of rotavirus variants with naturally occurring sequence duplications was greater than that of wild-type rotavirus [63].

### 3.6. Genetic Stability of rSA11 Rotaviruses Containing S Sequences

**Figure 7:**
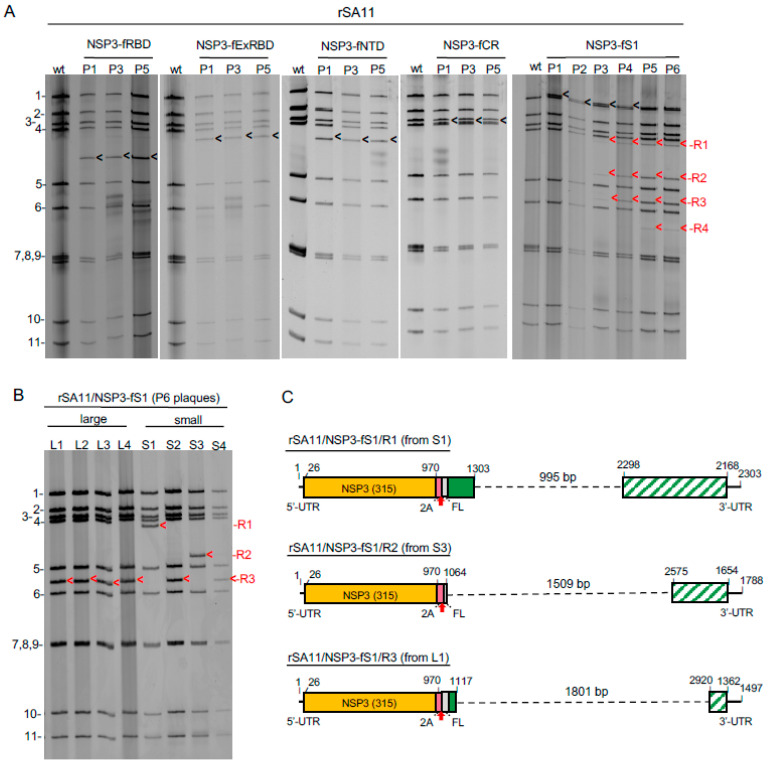
Genetic stability of rSA11 strains expressing SARS-CoV-2 S domains. rSA11 strains were serially passaged five to six times (P1 to P5 or P6) in MA104 cells. (**A**) Genomic RNAs were recovered from infected cell lysates and analyzed by gel electrophoresis. Positions of viral genome segments are labeled. Position of modified segment 7 (NSP3) dsRNAs introduced into rSA11 strains are denoted with black arrows. Genetic instability of the modified segment 7 (NSP3) dsRNA of rSA11/NSP3-fS1 yielded R1-R4 RNAs during serial passage. (**B**) Genomic RNAs prepared from large (L1–L4) and small (S1–S4) plaque isolates of P6 rSA11/NSP3-fS1. Segment 7 RNAs are identified as R1–R3, as in (**A**). (**C**) Organization of R1–R3 sequences determined by sequencing of segment 7 RNAs of L1, S1, and S3 plaque isolates. Sequence deletions are indicated with dashed lines. Regions of the S1 ORF that are no longer encoded by the R1–R3 segment 7 RNAs are indicated by slashed green-white boxes.

The genetic stability of the rSA11/NSP3-CoV2/S viruses were assessed by serial passage, with a fresh monolayer of MA104 cells infected with 1:1000 dilutions of cell lysates at each round. Electrophoretic analysis of the dsRNAs recovered from cells infected with rSA11/NSP3-fNTD, -fRBD, -ExRBD, or -ExCR showed no changes in the sizes of any of the 11 genome segments over 5 rounds of passage (P1–P5), including segment 7, indicating that these viruses were genetically stable (Figure 7A). In contrast, serial passage of rSA11/NSP3-S1 showed evidence of instability (Figure 7A). By the third round of passage, novel genome segments were appearing that were smaller than the 3.3 kbp segment 7 RNA. With continued passage, four novel segments (R1 to R4) became prominent and the 3.3 kbp segment 7 RNA was no longer detectable, suggesting that the high-passage virus pools (P3–P6) were populated by variants containing segment 7 RNAs derived from the 3.3-kb segment 7 RNA through internal sequence deletion. To evaluate this possibility, eight variants were recovered from the P6 virus pool by plaque isolation, four with a large (L) plaque phenotype and four with a small (S) plaque phenotype. Electrophoretic analysis of the genomes of the variants showed that none contained the 3.3 kbp segment 7 RNA (Figure 7B). Instead, six variants (L1, L2, L3, L4, S2, and S4) contained the R3 segment, and the other two variants contained either the R1 (S1) or R2 (R2) segment. No variants were recovered that contained the novel R4 segment.

Sequencing showed that the R1, R2, and R3 segments were in fact derivatives of the 3.3 kbp segment 7 RNA (Figure 7C). The R1, R2, and R3 RNAs all retained the complete 5′- and 3′-UTRs and NSP3 ORF of segment 7, but contained sequence deletions of 1.0 (R1), 1.5 (R2), or 1.8 (R3) kbp of S1 coding sequence. The fact that six of the eight variants isolated by plaque assay contained the R3 segment suggests that variants with this RNA may have a growth advantage over variants with the R1, R2, or R4 RNAs. Although genetic instability gave rise to rSA11/NSP3-fS1 variants lacking portions of the S1 ORF, none were identified that lacked portions of the NSP3 ORF. This suggests that NSP3 may be essential for virus replication, which would explain the failure of previous efforts by us to recover viable rSA11s encoding truncated forms of NSP3 through insertion of stop codons in the NSP3 ORF (data not shown). To gain a better understanding of the diversity of deletions introduced into the segment 7 (NSP3-fS1) RNA during serial passage, the total population of viral RNAs in high-passaged virus pools is being examined by direct RNA sequencing.

## 4. Summary

We have shown that reverse genetics can be used to generate recombinant rotaviruses that express, as separate products, portions of the SARS-CoV-2 S protein, including its immunodominant RBD. These results indicate that it may be possible to develop rotaviruses as vaccine expression vectors, providing a path for generating oral live-attenuated rotavirus-COVID-19 combination vaccines able to induce immunological protective responses against both rotavirus and SARS-CoV-2. Such combination vaccines would be designed for use in infants and young children and would allow the widespread distribution and administration of COVID-19-targeted vaccines by piggy backing onto current rotavirus immunization programs used in the USA and many other countries, both developed and developing. In addition, our findings raise the possibility that through the use of rotavirus as vaccine expression platforms, rotavirus-based combination vaccines could be made against other enteric viruses including norovirus, astrovirus, and hepatitis E virus.

We have determined that the 18.6 kbp rotavirus dsRNA can accommodate as much as 2.2 kbp of foreign sequence, which is sufficient to encode the SARS-CoV-2 S1 protein. However, in our hands, rSA11s encoding S1 were not genetically stable and failed to express the appropriate S1 product, for reasons that are uncertain but under further investigation. Rotaviruses carrying large amounts of foreign sequence are characteristically genetically unstable (this study and data not shown), but those with foreign sequences of <1.0–1.5 kbp are stable over 5–10 rounds of serial passage at low MOI and, thus, can be developed into vaccine candidates. The coding capacity provided by 1.0–1.5 kbp of extra sequence is sufficient to produce recombinant rotaviruses that encode the SARS-CoV-2 NTD, RBD, or S2 core along with trafficking signals that can promote engagement of S products with antigen-presenting cells and naive B-lymphocytes. Current work is underway to gain insight into how successful rotaviruses expressing SARS-CoV-2 products are in inducing neutralizing antibodies in immunized animals.

## Figures and Tables

**Table 1:** Primers used to produce pT7/NSP3-2A-CoV2 plasmids.

Primer	Sequence
Vector_For	TGACCATTTTGATACATGTTGAACAATCAAATACAG
Vector_Rev	GCTAGCCTTGTCATCGTCATCCT
NTD_For	GATGACAAGGCTAGCTGTGTTAATCTTACAACCAGAACTCAATTACCCC
NTD_Rev	GTATCAAAATGGTCAGTCAAGTGCACAGTCTACAGCATC
ExRBD_For	GATGACAAGGCTAGCGGAATCTATCAAACTTCTAACTTTAGAGTCCAACCA
ExRBD_Rev	GTATCAAAATGGTCATGTTATAACACTGACACCACCAAAAGAACA
RBD_For	GATGACAAGGCTAGCTTGTGCCCTTTTGGTGAAGTTT
RBD_Rev	GTATCAAAATGGTCAAGTTGCTGGTGCATGTAGAAGT
CR_For	GATGACAAGGCTAGCTCTATTGCCATACCCACAAATTTTACTATTAGTGT
CR_Rev	GTATCAAAATGGTCAAGTTGTGAAGTTCTTTTCTTGTGCAGG
S1_For	GATGACAAGGCTAGCGTGTTTGTTTTTCTTGTTTTATTGCCACTAGTCT
S1_Rev	GTATCAAAATGGTCAACGTGCCCGCCG

**Table 2:** Properties of recombinant rRV/NSP3-2A-CoV2 strains.

Virus Strain			Genome Segment 7					
			RNA (bp)	Protein Product				NCBI Accession #
Abbreviated Name	Formal Name *	Genome Size/Increase Over wt (bp)		Uncleaved (aa)	2A Cleaved (aa)	Uncleaved (kDa)	2A Cleaved (kDa)	
rSA11/wt	RVA/Simian-lab/USA/SA11wt/2019/G3P[2]	18,559/0	1105	315	nd	36.4	nd	LC178572
rSA11/NSP3-fNTD	RVA/Simian-lab/USA/SA11(NSP3-P2A-CoV2:fNTD)/2020/G3P[2]	19,537/978	2083	641	336 + 305	73.2	38.5 + 34.8	MW059024
rSA11/NSP3-fRBD	RVA/Simian-lab/USA/SA11(NSP3-P2A-CoV2:fRBD)/2020/G3P[2]	19,264/705	1810	550	336 + 214	62.7	38.5 + 24.3	MT655947
rSA11/NSP3-fExRBD	RVA/Simian-lab/USA/SA11(NSP3-P2A-CoV2:fExRBD)/2020/G3P[2]	19,564/1005	2110	650	336 + 314	74.7	38.5 + 35.2	MT655946
rSA11/NSP3-fCR	RVA/Simian-lab/USA/SA11(NSP3-P2A-CoV2:fCR)/2020/G3P[2]	19,798/1239	2344	728	336 + 392	81.4	38.5 + 42.9	MW059025
rSA11/NSP3-fS1	RVA/Simian-lab/USA/SA11(NSP3-P2A-CoV2:fS1)/2020/G3P[2]	20,752/2193	3298	1046	336 + 710	118.1	38.5 + 79.6	MW059026
rSA11/NSP3-fS1/R1	RVA/Simian-lab/USA/SA11(NSP3-P2A-CoV2:fS1/R1)/2020/G3P[2]	19,757/1198	2303	431	336 + 95	49.6	38.5 + 11.1	MW353715
rSA11/NSP3-fS1/R2	RVA/Simian-lab/USA/SA11(NSP3-P2A-CoV2:fS1/R2)/2020/G3P[2]	19,333/683	1788	367	336 + 31	42.1	38.5 + 3.7	MW353716
rSA11/NSP3-fS1/R3	RVA/Simian-lab/USA/SA11(NSP3-P2A-CoV2:fS1R3)/2020/G3P[2]	18,951/392	1497	410	336 + 74	47.2	38.5 + 8.8	MW353717

* Formal strain names were assigned according to Matthijnssens et al. [48]. nd: not determined, no 2A cleavage site present; wt: wild type.

## Data Availability

Data is contained within the article. Sequences of recombinant viruses were deposited in NCBI GenBank {https://www.ncbi.nlm.nih.gov/genbank/}, with accession numbers provided in Table 1. Additional data related to this paper may be requested from the authors.
